# Predictors of Thirty-day Mortality and Length of Stay in Operative Subdural Hematomas

**DOI:** 10.7759/cureus.5657

**Published:** 2019-09-14

**Authors:** Tyler Ball, Brent G Oxford, Ahmad Alhourani, Beatrice Ugiliweneza, Brian J Williams

**Affiliations:** 1 Neurological Surgery, University of Louisville School of Medicine, Louisville, USA

**Keywords:** subdural hematoma, traumatic brain injury, outcome prediction, head injury, coagulation, traumatic head injury, neurosurgery, national surgical quality improvement program

## Abstract

The rate of postoperative morbidity and mortality after subdural hematoma (SDH) evacuation is high. The aim of this study was to compare mortality statistics from a high-volume database to historical figures and determine the most significant preoperative predictors of mortality and length of stay (LOS). The National Surgical Quality Improvement Program registry was searched (2005-2016) for patients with operatively treated SDHs, of which 2709 were identified for univariate analysis. After exclusion for missing data, 2010 individuals were analyzed with multivariable logistic regression. Primary outcome was 30-day mortality. The average patient age was 68.8 ± 14.9 years, and 64.1% were males. Upon multivariate analysis, nine variables were found to be associated with increased mortality: platelet count < 135,000 (OR 2.04, 95% CI 1.39-2.99), INR >1.2 (OR 1.87, 95% CI 1.34-2.6), bleeding disorder (OR 1.80, 95% CI 1.32-2.46), need for dialysis within two weeks preoperatively (OR 5.69, 95% CI 3.15-10.27), ventilator dependence in the 48 hours preceding surgery (OR 3.99, 95% CI 2.82-5.63), disseminated cancer (OR 2.95, 95% CI 1.34-6.47), WBC count >10,000 (OR 1.55, 95% CI 1.15-2.08), totally dependent functional status (OR 1.84, 95% CI 1.2-2.8), and each increasing year of age (OR 1.04, 95% CI 1.031-1.05). It is not surprising that chronic conditions and functional status were associated with increased mortality. However, specific laboratory abnormalities were also associated with increased mortality at levels generally considered within normal limits. More studies are needed to determine if correcting lab abnormalities preoperatively can improve outcomes in patients with intrinsic coagulopathy.

## Introduction

Subdural hematomas (SDHs) are one of the most common pathologies managed by neurosurgeons. Many patients with SDHs require surgical intervention [1]. However, the aging population and increased usage of antiplatelet and anticoagulant medications complicate the clinical decision making for surgical intervention. Therefore, prognostication and management of patients with SDHs remain pertinent to modern practice.

Historically, mortality among patients with traumatic acute SDHs treated surgically is high, with a range of 30%-70% reported [2-9]. More recent studies have demonstrated a 12%-15% mortality rate for SDHs overall [1, 10-11]. Many survivors also suffer from reduced functional capacity and disability even with prompt treatment. The dilemma for neurosurgeons is attempting to decide preoperatively who can achieve a “good outcome” with surgery. Current literature has established advanced age, low Glasgow Coma Scale (GCS) at admission, and SDH location as factors that influence mortality among SDH patients [2, 7, 8, 12-14]. Additional prognostic factors of mortality include midline shift (MLS), patency of cisterns, and hematoma volume [2,15-17]. The goal in writing this paper was to compare current mortality statistics from a high volume database to historical figures, and to determine the most significant preoperative predictors of mortality.

## Materials and methods

We performed a retrospective review using the American College of Surgeons National Surgical Quality Improvement Program (ACS NSQIP) database. The ACS NSQIP maintains a prospectively collected database from over 600 participating sites and includes perioperative, intraoperative, and 30-day postoperative variables for a wide variety of surgical procedures [18]. An inter-rater reliability audit of participating sites is employed to ensure high quality data. The ACS NSQIP database has been previously discussed and validated in the surgical literature [19-20]. This study was conducted in adherence to the NSQIP participant data use agreement.

We queried the NSQIP database from 2005 to 2016 using the CPT code 61312 (crainectomy or craniotomy for evacuation of supratentorial hematoma: extradural or subdural) and ICD-9 code (432.1, 852.2, 852.3) and ICD-10 codes (S06.5X, I62) to identify cases of isolated supratentorial SDHs. We identified 2709 cases that met these criteria.

A 30-day mortality was used as the primary outcome. Hospital length of stay (LOS) was a secondary outcome. All variables related to demographics, medical comorbidities, perioperative factors and laboratory results that showed variability were extracted. Standard laboratory cutoffs were used to convert the values into categorical values. Time from admission to operating room was categorized into same day or after.

Statistical analysis

Statistical analysis was performed using MATLAB and Statistics Toolbox Release 2017b (The MathWorks, Inc., Natick, Massachusetts, USA) and SPSS (IBM Corp., Armonk, New York, USA).

For univariate analysis, Student’s t-tests were used for continuous variables and reported with mean and standard deviation while Pearson χ2 tests were used for categorical variables and reported as number and percentage per group. A multivariate logistic regression model for predicting mortality was fit using all significant variables on univariate analysis. A separate multivariate logistic regression model was attempted for return to the operating room. For LOS, the values were log transformed to convert the distribution into a normal distribution. LOS was treated as a continuous variable and a multivariate linear regression was fit using forward stepwise addition based on the effect of the model deviance using variables with significant effects on univariate analysis.

To identify the optimal cutoffs for preadmission laboratory values including the international normalized ratio (INR) and platelet count for mortality prediction, separate multivariate logistic regression models were fit while iteratively changing the cutoff across the range of available values. The model was optimized based off of the information criteria. See Appendix A for more detailed statistical methodology.

The ACS NSQIP and the hospitals participating in the ACS NSQIP are the source of the data used herein; they have not verified and are not responsible for the statistical validity of the data analysis or the conclusions derived by the authors.

## Results

Patient demographics and comorbidities

A total of 2709 individuals who underwent surgical evacuation of SDHs between 2005 and 2016 were selected from the ACS NSQIP database. There were 699 patients excluded from the multivariate analysis due to absence of significant data, leaving 2010 available for the prediction models.

 The average patient age (± SD) was 68.8 ± 14.9 years. A disproportionate number of men were affected, 64.1% of included patients, versus 35.9% women. Most patients (80.0%) were considered functionally independent prior to surgery. Among functionally impaired patients, 11.9% were considered partially dependent and 8.2% were considered totally dependent before surgical intervention. See Table 1 for a summary of patient demographics and Table 2 for a summary of mortality rates in patients with various comorbidities.

**Table 1:** Patient demographics.

Table 1. Summary of patient demographics			
Variable	No	(%)
Total number of patients	2709	100
Overall mortality	450	16.61
Sex		
Male	1736	64.11
Female	972	35.89
Race		
American Indian or Alaska native	27	1.15
Asian	111	4.73
Black or African American	348	14.83
Hispanic	216	9.08
Native Hawaiian or Pacific Islander	9	0.38
White	1639	69.83
ASA Class		
1	15	0.56
2	191	7.08
3	1172	43.42
4	1123	41.61
5	198	7.34
	Mean	SD
Age (years)	68.83	14.87
Height (inches)	66.84	4.24
Weight (lbs)	172.25	45.92
BMI	27.63	6.48

**Table 2:** Summary of mortality on univariate analysis. *Bleeding disorder defined as: patients with any chronic, persistent, active condition that places the patient at risk for excessive bleeding (e.g., vitamin K deficiency, hemophilia, thrombocytopenia, chronic anticoagulation therapy that has not been discontinued prior to surgery), and patients with active heparin-induced thrombocytopenia (HIT), and patients who has a past medical history of thrombocytopenia and a low platelet count at the time of the principal operative procedure. The following cases are not included: patient on chronic aspirin therapy; patient on nonsteroidal anti-inflammatory drugs (NSAIDs); When medications are prescribed for prophylactic use, for the principal operative procedure only; patient with a history of HIT in the past which is not deemed active.

Table 2. Summary of mortality univariate analysis						
Variable	No	(%)	Mortality (%)	Chi²	p-Value
Diabetes					
None	2115	78.07	15.18	14.70	0.0006
Insulin	236	8.71	22.88		
Noninsulin	358	13.22	21.32		
Dyspnea					
None	2584	95.39	16.06	21.57	<0.0001
At rest	46	1.70	41.3		
With moderate exertion	79	2.92	20.25		
Functional status					
Independent	2130	78.63	14.27	67.10	<0.0001
Partially dependent	316	11.66	18.04		
Completely dependent	218	8.05	35.78		
Sepsis					
None	2252	83.25	13.81	75.47	<0.0001
SIRS	409	15.12	30.32		
Sepsis	32	1.18	33.33		
Septic shock	12	0.44	41.66		
ASA Class					
1	15	0.56	0	312.45	<0.0001
2	191	7.08	2.62		
3	1172	43.42	6.66		
4	1123	41.61	23.78		
5	198	7.34	50.00		
Ascites	13	0.48	53.85	13.07	0.0003
*Bleeding disorder	673	24.84	31.8	149.09	<0.0001
CHF	85	3.14	32.94	16.89	<0.0001
COPD	150	5.54	20.66	1.89	0.171
Dialysis	91	3.36	45.05	55.00	<0.0001
Disseminated cancer	58	2.14	37.93	19.45	<0.0001
Hypertension	1744	64.38	23.25	17.95	<0.0001
Open wound	98	3.62	24.49	4.56	0.0328
Renal failure	21	0.78	38.1	7.05	0.0079
Steroids administered	79	2.92	24.05	3.25	0.0714
Transfusion within 72 hours pre-op	62	2.29	27.42	5.35	0.0207
Weight loss >10% over 6 months	46	1.70	30.43	6.46	0.0111

Prediction of mortality

**Figure 1:**
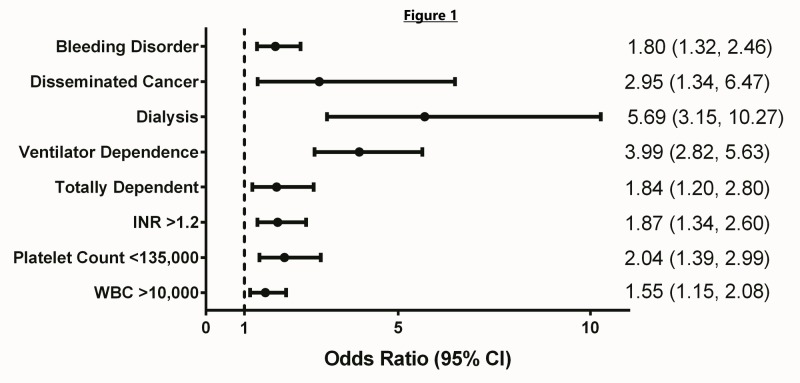
Predictors of mortality. All of the above were included as dichotomous variables on multivariate analysis and found to be statistically significant predictors of mortality. Ventilator dependence was defined as the patient requiring ventilator-assisted respiration at any time during the 48 hours preceding surgery. Patients who were “totally dependent” required total assistance with all ADLs. “Bleeding disorder” includes patients with any chronic, persistent, active condition that places the patient at risk for excessive bleeding (e.g., vitamin K deficiency, hemophilia, thrombocytopenia, chronic anticoagulation therapy that has not been discontinued prior to surgery).

First, we defined the optimal cutoff for INR and platelet count that best dichotomized outcomes. Interestingly, an INR >1.2 and a platelet count <135 were the optimal values based on Bayesian information criterion (BIC) and Akaike information criterion (AIC). Next, we used multivariate analysis that found several significant factors related to mortality. Of note, the need for dialysis within two weeks prior to surgery showed the greatest increase in risk of mortality (OR = 5.69, 95% CI 3.15-10.27) followed by ventilator dependence in the 48 hours preceding surgery (OR = 3.99, 95% CI 2.82-5.63) and disseminated cancer (OR 2.95, CI 1.34-6.47). INR>1.2, platelet count less than 135,000, bleeding disorders (defined in Figure 1 caption), WBC >10, increasing age, and a totally dependent functional status were also associated with an increased mortality in the multivariate analysis. See Table 3 for a summary of the regression model. See Figure 1 for a comparison of some of the most significant predictors of mortality.

**Table 3:** Regression model for mortality. Omnibus test of model coefficients: Chi-square = 518.77, df = 16, p = 0. Hosmer and Lemeshow Test: Chi-square = 11.897, df = 8, p = 0.156.

Table 3. Regression model for mortality								
Variable		Beta coefficient	p-value		Relative risk		95% C.I.for EXP(B)	
						Lower		Upper
Constant		-4.58	0	0.01				
WBC	>10	0.436	0.004	1.546		1.149		2.081
Platelet count	<135	0.713	0	2.04		1.393		2.988
INR	>1.2	0.624	0	1.866		1.339		2.6
Age (years)		0.042	0	1.043		1.031		1.055
Functional status	Independent (reference)	-	-	-		-		-
	Partially dependent	0.366	0.075	1.442		0.964		2.156
	Totally dependent	0.608	0.005	1.837		1.204		2.802
Ventilator		1.383	0	3.987		2.824		5.629
Dialysis		1.738	0	5.689		3.151		10.271
Disseminated cancer		1.081	0.007	2.947		1.342		6.474
Bleeding disorder		0.589	0	1.803		1.323		2.457
ASA Class	5-Moribund (reference)	-	-	-		-		-
	2-Mild disturb	-2.618	0.001	0.073		0.017		0.319
	3-Severe disturb	-1.608	0	0.2		0.121		0.332
	4-Life threat	-0.832	0	0.435		0.282		0.672
Days to operation		-0.354	0.02	0.702		0.521		0.945
Return to OR		-0.67	0.002	0.512		0.333		0.787

 

 

**Figure 2:**
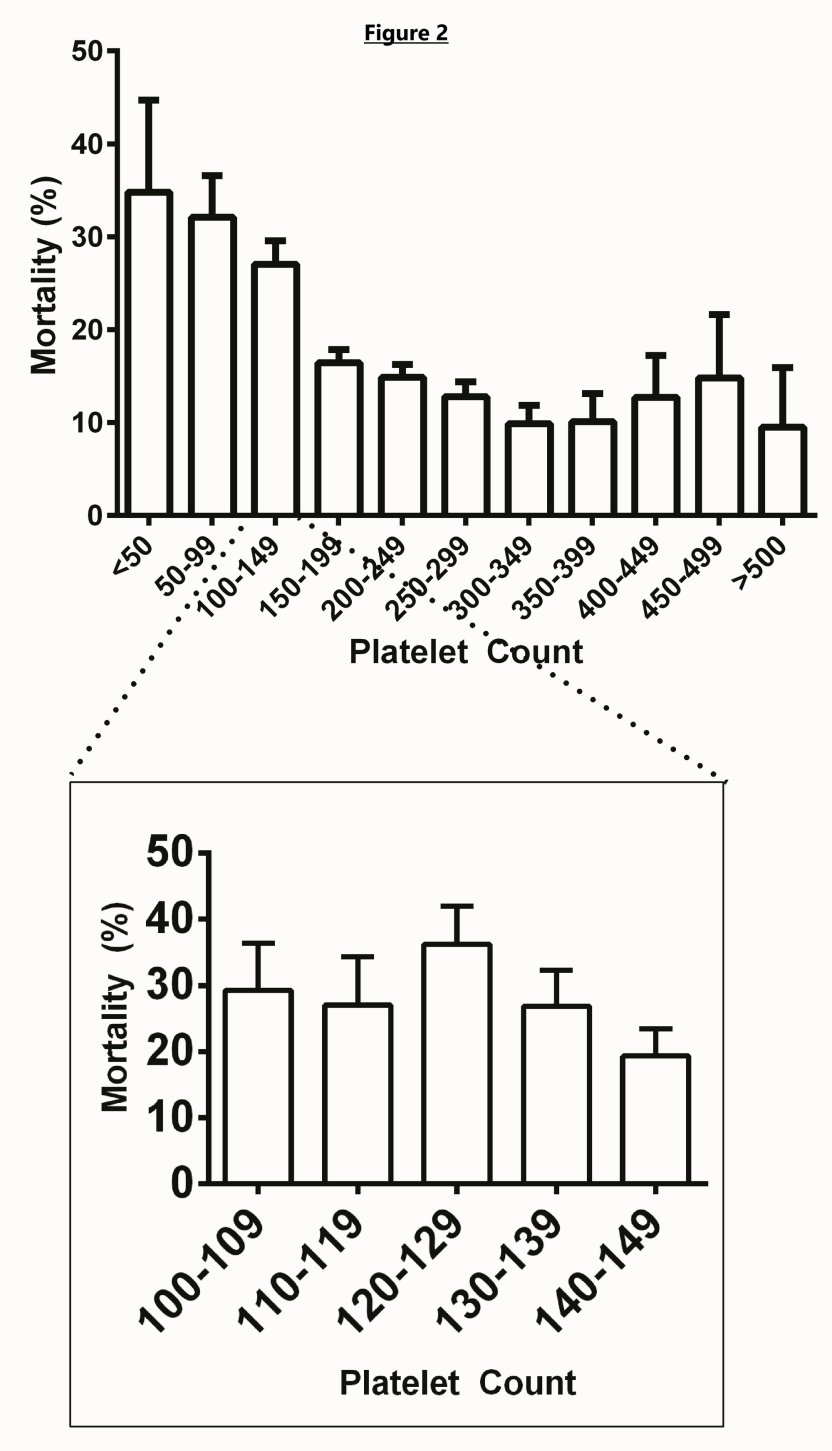
Mortality compared to platelet count. Platelet count reported in thousands. Error bars reflect standard error of the means.

Platelet count <135,000 was strongly associated with mortality in both the univariate and multivariate models (OR = 2.04, 95% CI 1.39-2.99). Patients with a platelet count <135,000 had a 32.1% mortality, whereas patients with a platelet count greater than 135,000 had a mortality of only 14.4%. Patients with a platelet count of >135,000 were also shown to be associated with a decreased LOS (β = 0.03, p < 0.001). Figure 2 shows the association between platelet count and mortality.

**Figure 3:**
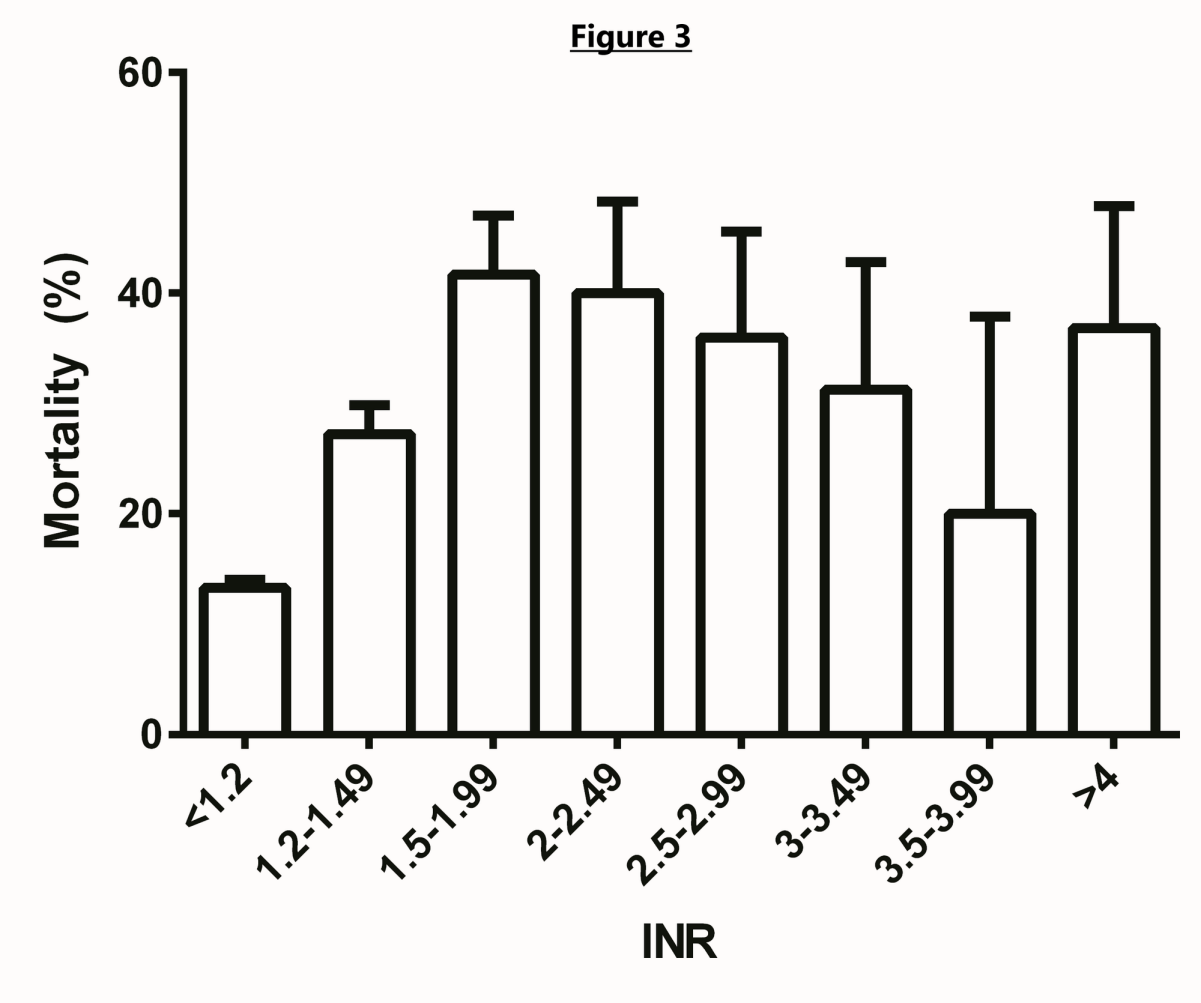
Comparison between INR and mortality. Error bars reflect standard error of the means.

**Figure 4:**
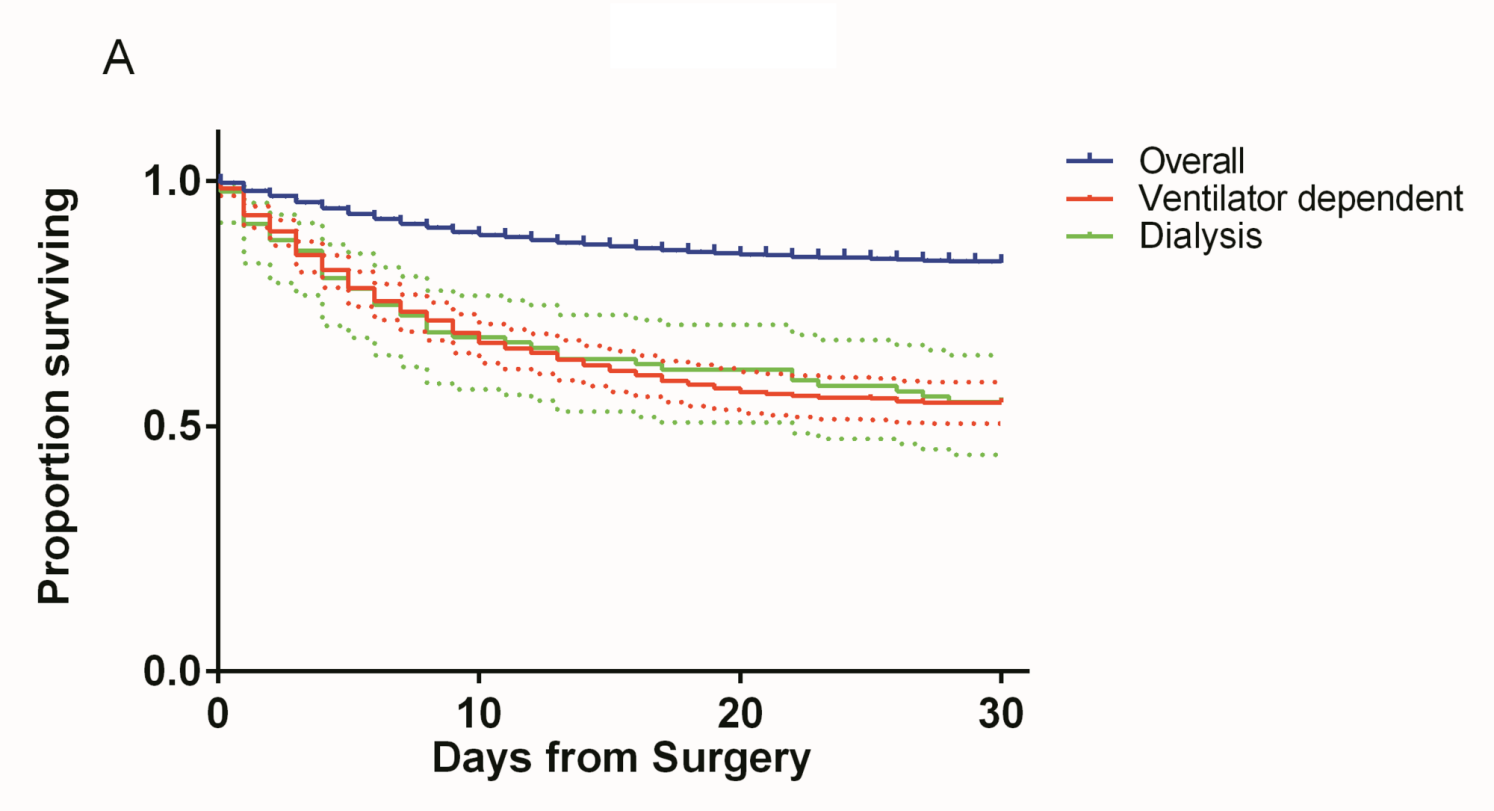
Kaplan-Meier curves showing overall 30-day survival compared to 30-day survival in ventilator dependent and dialysis patients with 95% confidence intervals.

**Figure 5:**
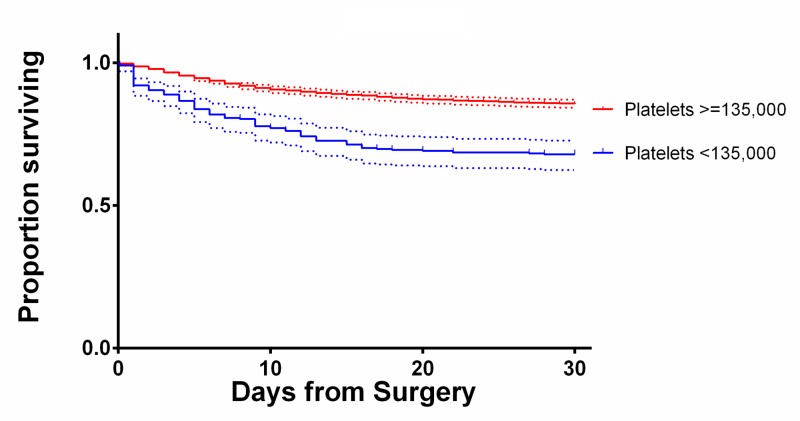
Kaplan-Meier curves showing 30-day survival of patients with platelet counts above and below the 135,000 cutoff with 95% confidence intervals.

**Figure 6:**
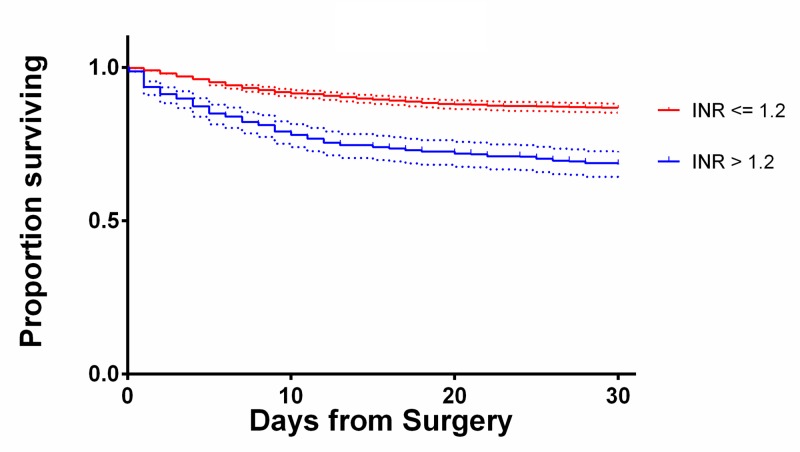
Kaplan-Meier curves showing 30-day survival of patients with INR above and below the 1.2 cutoff with 95% confidence intervals.

The INR showed a positive association with mortality. Average INR among survivors was 1.16 ± 0.45 and among expired patients was 1.34 ± 0.65. Patients with an INR <=1.2 showed a 13.3% mortality, significantly lower than the 31.6% mortality for patients with an INR >1.2. Using an INR of 1.2 as a cutoff in the multivariate model demonstrated increased INR was associated with increased mortality (OR = 1.87, 95% CI 1.34-2.60). INR greater than 1.2 was also associated with increased LOS (β = 0.02, p < 0.0069). Figure 3 shows the relationship between INR and mortality. The 30-day Kaplan Meier survival curves for selected significant variables are shown in Figures 4-6.

**Figure 7:**
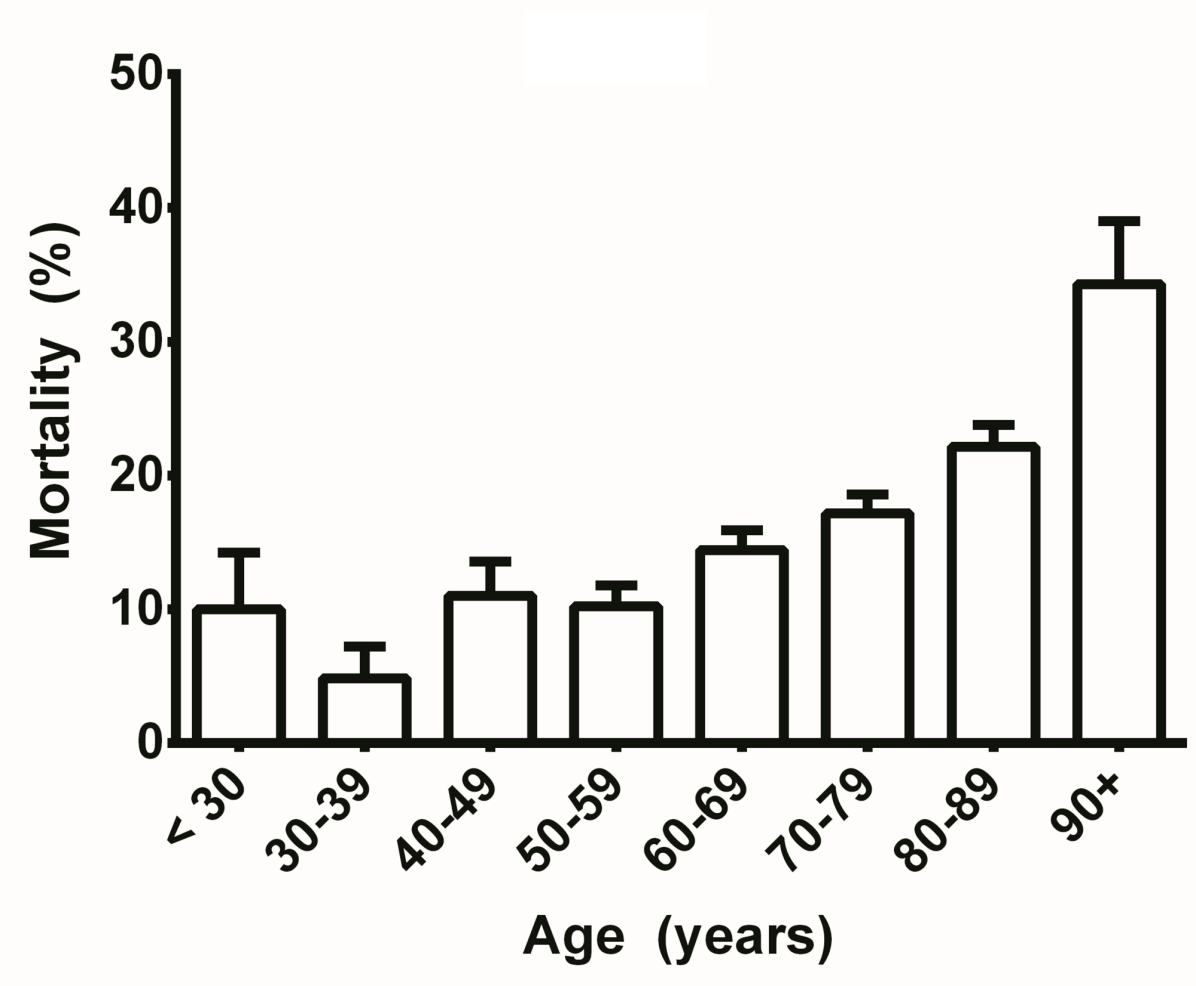
Univariate analysis of mortality by age. Error bars reflect standard error of the means.

Every year of increasing age was associated with a small but significant incremental increase in mortality as demonstrated by the positive beta coefficient (OR 1.043, CI 1.031-1.055, Table 3). See Figure 7 for a breakdown of mortality by age broken into decades.

Length of stay

The average LOS (± SD) was 10.4 ± 13.1 days among all patients. Patients discharged to skilled care, acute care, or rehab had an average LOS of 13.8 ± 11.2 days. Slightly less than half (48.8%) of the patients stayed one week or less. The longest hospitalization was 112 days, but greater than 90% of patients stayed less than three weeks.

 A logistic regression model for LOS showed WBC, platelet count, INR, functional status, ventilator use, dialysis, operation time, sepsis, ASA class, days from admission until operation, and need to return to the operating room as significant predictors. See Table 4 for a summary.

**Table 4:** Logistic regression model for hospital length of stay. Model evaluation: R-squared = 0.214, F-stat = 29.9, p = 1.23E-75.

Table 4. Logistic regression model for hospital length of stay					
Variable		Estimate	SE	tStat	p-Value
(Intercept)		1.18	0.01	79.24	0.0000
WBC	>10	0.02	0.01	3.79	0.0002
Platelet count	<135	0.03	0.01	3.64	0.0003
INR	>1.2	0.02	0.01	2.71	0.0069
Functional status	Independent (reference)	-	-	-	-
	Partially dependent	0.02	0.01	2.80	0.0052
	Totally dependent	0.03	0.01	3.16	0.0016
Ventilator		0.05	0.01	5.93	0.0000
Operation time		0.00	0.00	6.36	0.0000
Dialysis		0.05	0.02	2.90	0.0038
Sepsis	None (reference)	-	-	-	-
	Sepsis	0.05	0.03	2.10	0.0363
ASA Class	5-Moribund (reference)	-	-	-	-
	1-No disturb	-0.10	0.04	-2.73	0.0063
	2-Mild disturb	-0.07	0.02	-4.43	0.0000
	3-Severe disturb	-0.05	0.01	-3.80	0.0001
	4-Life threat	-0.03	0.01	-1.97	0.0485
Days from admission to operation		0.05	0.01	9.40	0.0000
Return to OR		0.06	0.01	8.23	0.0000

## Discussion

This review of the NSQIP database showed a similar overall mortality rate (16.5%) of patients with surgically treated SDHs compared to that found by Lukasiewicz in 2016 (18%) [21]. Similarly, increasing age, higher ASA class, and bleeding disorders were associated with higher mortality. Additional factors in the current study that were significantly correlated with mortality were INR>1.2, platelet count <135,000, dialysis dependence, ventilator dependence within 48 hours prior to surgery, disseminated cancer, totally dependent functional status, and WBC >10,000.

While this retrospective review utilized the same data source as Lukasiewicz et al., the database now contains laboratory information and clinical variables not previously reported. Additionally, sample size for the current study is over four times larger than was available for the previous study. This adds strength to the congruent findings and sheds light on clinically relevant variables that were not available for the prior study.

 The prior study reported that “bleeding disorders” were associated with increased mortality. “Bleeding disorder” is a broad term that can apply to a number of different pathological or pharmacologically induced bleeding diatheses. However, the label is of limited clinical utility, from both prognostic and therapeutic standpoints. More specific parameters, i.e., platelet count and INR, can give clinicians more quantifiable information preoperatively. Both were shown to have an association with increased mortality. In contrast, Senft et al. have shown that patients on oral anticoagulants with SDHs who had their pharmacologic coagulopathy reversed in a timely manner did not differ significantly in terms of overall outcome when compared to historical cohorts [22]. One interpretation of this discrepancy could be that thrombocytopenia may be a marker of worse overall health that is not affected by giving platelets, as opposed to aspirin-induced platelet dysfunction that can be partially overcome by platelet transfusion. Likewise, an elevated INR can be the result of taking anticoagulants (i.e., coumadin), or as a surrogate marker for poor liver function. Patients’ use of coumadin was not divulged in this dataset, prohibiting direct comparison between the groups.

Thrombocytopenia can be seen in patients with poor renal function [23]. However, as can be seen on the Kaplan-Meier curves in Figure 4, the mortality of patients who were dialysis dependent was greater than that of patients with isolated thrombocytopenia. In fact, dialysis dependence had the highest OR for mortality (OR 5.69, 95% CI 3.15-10.27) on multivariate analysis.

Additionally, ventilator dependence within the 48-hour period preoperatively was associated with an OR for mortality of 3.99 (CI 2.82-5.63). The reason for mechanical ventilation was not disclosed in the dataset. As patients with severe traumatic brain injury (TBI) are generally intubated for airway protection, it is possible that many of the ventilated patients also had more severe head injuries that contributed to higher mortality. However, GCS was not available for this dataset. 

Disseminated cancer had an OR of 2.95 (CI 1.34-6.47) for mortality, supporting the evidence that the patient’s general medical condition has a large impact on mortality.

Age was found to have a small but significant effect on mortality. Every increasing year of age was associated with an incremental increase in the risk of mortality (OR 1.04, 95% CI 1.031-1.05).

It has been established in the literature that SDHs have a high associated morbidity [2-9]. However, larger collections of retrospective data on this patient population are allowing us to determine variables that make patients with SDHs more likely to have a poor outcome. Carefully analyzing these data may aid clinicians in deciding which patients may benefit from operative intervention.

There are several potential limitations of this study. First, the data were collected retrospectively. Second, there was no indication of the severity of the TBI based off clinical exam. Also, anticoagulant and antiplatelet use were not reported. Additionally, for patients with an elevated INR, the use of reversal agents, the types of reversal agents used, and the timing of reversal for anticoagulation were not available. Finally, mechanism of injury was not available in this dataset. One would expect higher mortality in the subset of SDHs secondary to high speed motor vehicle accidents (MVAs) or high impact blunt trauma that often cause SDHs in the younger population, given the underlying brain injury. Alternatively, lower velocity trauma like ground level falls that are often responsible for SDHs in the older population often have little underlying parenchymal injury. However, even in a cohort of only high velocity trauma, it would be difficult to control for the differing degrees of brain injury.

## Conclusions

This study showed that general medical conditions and preoperative functional status were associated with increased mortality and LOS in patients with surgically treated SDHs, as expected. However, specific laboratory abnormalities (platelet count <135,000, INR > 1.2) were also shown to contribute to increased mortality, and the quantitative values at which coagulopathy and thrombocytopenia were shown to be significantly associated with mortality were in a range that many hospital laboratories consider within normal limits. More studies are needed to determine if correcting lab abnormalities preoperatively can improve outcomes in patients with intrinsic coagulopathy (i.e., not due to an anticoagulant medication).

## Appendices

Appendix A

Regarding the multivariate analysis for mortality, variables were included using backwards stepwise elimination based on the effect of the model deviance to prevent overfitting the model. Final model fit was assessed with Omnibus test of model coefficient and Hosmer and Lemeshow Test for goodness of fit.

Regarding determination of the optimal cutoff values for INR and platelet count, the BIC and AIC were extracted for each model. The model with the smallest AIC and BIC represents the best fit and the cutoff was determined as the optimal cutoff for dichotomizing the variable.
